# First person – Anjali Bajpai

**DOI:** 10.1242/dmm.046136

**Published:** 2020-07-24

## Abstract

First Person is a series of interviews with the first authors of a selection of papers published in Disease Models & Mechanisms, helping early-career researchers promote themselves alongside their papers. Anjali Bajpai is first author on ‘A *Drosophila* model of oral peptide therapeutics for adult intestinal stem cell tumors’, published in DMM. Anjali is a Wellcome Trust DBT India Alliance early-career fellow in the lab of Prof. Pradip Sinha at Indian Institute of Technology, Kanpur, India, investigating the developmental principles that govern carcinogenesis using *Drosophila* as a model system.


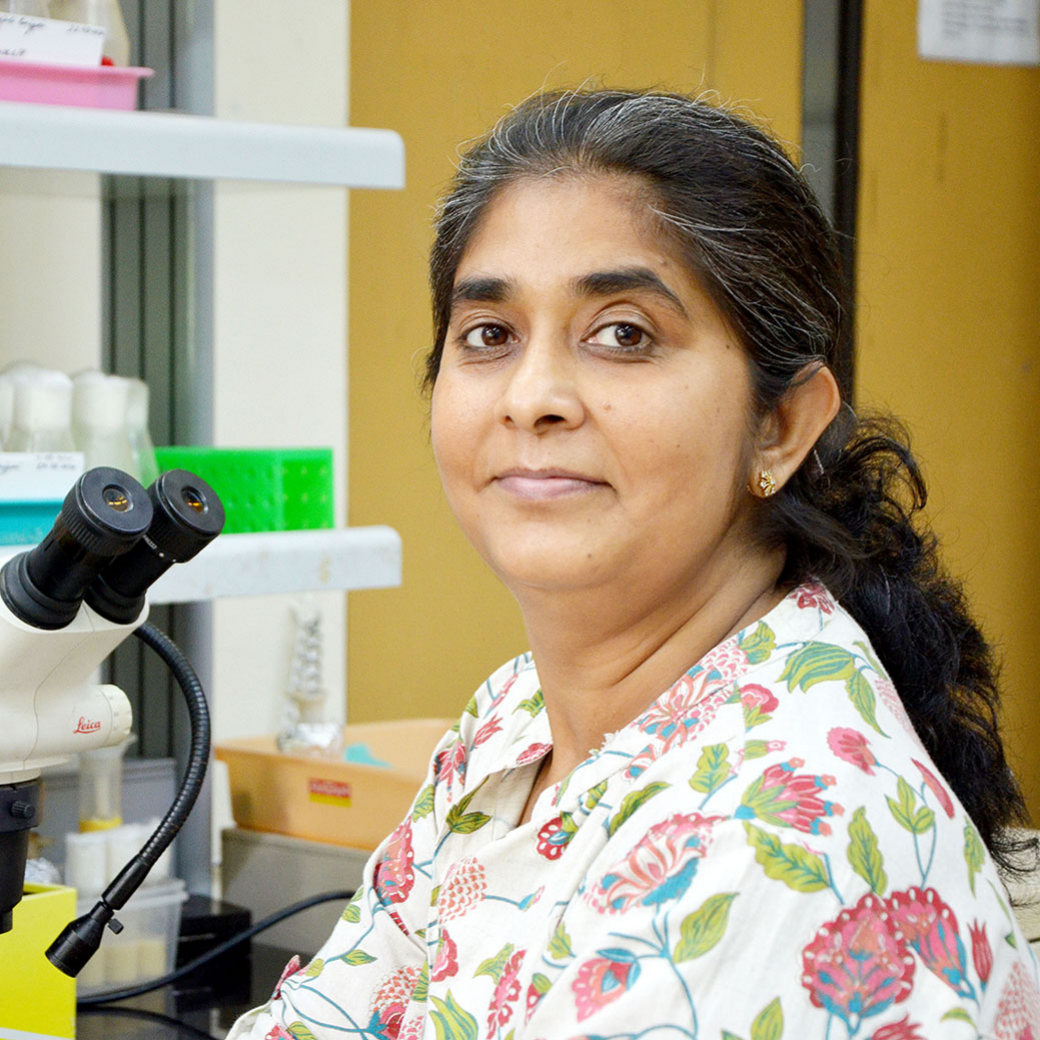


**Anjali Bajpai**

**How would you explain the main findings of your paper to non-scientific family and friends?**

Despite the many great advances in cancer therapy, there is a constant need to develop newer drugs with minimal off-target effects; however, cancer drug discovery is a long-drawn process. In recent years, the tiny, yet genetically highly tractable, model organism the fruit fly, *Drosophila*, has offered several new options of identifying novel cancer targets and feasibility of screening potential anti-cancer drugs. This is possible since *Drosophila* and humans display striking similarity at cellular and molecular levels. In this study, we show that genetically induced intestinal tumors in adult fruit flies can be arrested by feeding these tumor-bearing flies with a ‘peptide’ drug, which is derived from a protein that is naturally present in the fruit fly (and also in humans). We have demonstrated that our peptide drug arrests cell proliferation by binding to and inhibiting growth-stimulating cellular oncoproteins. Notably, this peptide also arrests the growth of human colorectal and prostate cancer cells in a series of cell-based assays.

**What are the potential implications of these results for your field of research?**

The implications of this study are twofold. While *Drosophila* has largely been used to screen small-molecule compounds, our study shows that it could be as effective to screen large biomolecules, such as the 26-amino-acid peptide used in this study. This is noteworthy, since peptide-based therapeutics hold much promise specifically to target large ‘undruggable’ biomolecules, such as transcription factors with bulky interacting surfaces, often not amenable to pharmacological inhibition using small-molecule drugs. However, the challenge lies in design and high-throughput screen of peptide-based drugs against a plethora of possible cellular targets. It is here that the fruit fly could prove extremely valuable. *Drosophila* as an *in vivo* system not only offers rapid assessment of drug action but also allows real-time assessment of tumor growth, invasion and metastasis.

**What are the main advantages and drawbacks of the model system you have used as it relates to the disease you are investigating?**

Fruit fly offers many advantages as a cancer model system, most important being its recapitulation of the major hallmarks of human cancers. Interestingly, many of the human oncogenes were first identified in the fruit fly. Further, the ease of genetic manipulation in this model organism allows one to dissect out the molecular mechanism of tumorigenesis and examine the efficacy of the therapeutic molecule under study. On the other hand, a drawback on the use of fruit fly as a cancer model is that even a single oncogenic ‘hit’ can give rise to aggressive tumors, which is mostly not the case in humans, with few exceptions. Hence, the low molecular redundancy in flies, while advantageous in dissecting out intricate signaling networks; extrapolations of the insights obtained from fly to humans require studied caution.

“[…] we were amazed and pleasantly surprised to see arrest of intestinal stem cell tumors in flies fed the TONDU peptide.”

**What has surprised you the most while conducting your research?**

Our study was hypothesis driven following our earlier observation of inhibition of neoplastic tumors by the endogenous fly protein, Vestigial. Further, TONDU peptide was shown to inhibit tumors when injected in mouse xenograft models. Therefore, we had sound reasons to believe that it would work in fly, except that we were testing a previously untested route of delivery, namely oral uptake of the peptide. Indeed, we were amazed and pleasantly surprised to see arrest of intestinal stem cell tumors in flies fed the TONDU peptide. This is really significant since I believe it opens up an entire field of peptide-based therapeutics to be explored in flies, and examine its oral route of administration in higher model organisms.

**Describe what you think is the most significant challenge impacting your research at this time and how will this be addressed over the next 10 years?**

The fruit fly *Drosophila* has proven to be invaluable to uncovering many basic tenets of cancer biology besides being a remarkable *in vivo* platform for drug discovery and screening. Thus, while the fruit fly is well poised to be explored for biomedical translational research, the flux of information and knowledge sharing between biologists, clinicians and pharmacologists needs to be further strengthened and encouraged to facilitate impactful translational research goals. For instance, one of the open challenges in cancer is to understand the role of host genetics in cancer initiation, progression and distant metastasis. It remains to be fully understood how genetic variations among individuals determine the course of cancer progression, which has a direct bearing upon how individuals respond to cancer therapy. It is here that the amazing power of *Drosophila* genetics should be explored to its fullest.

**Figure:**
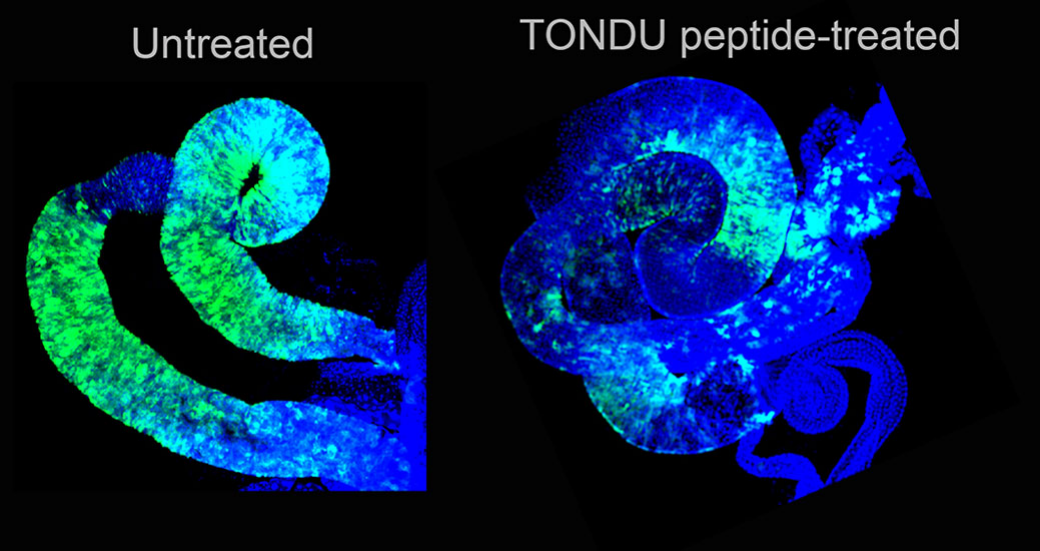
**Oral peptide-mediated inhibition of gut tumors in fruit fly.** Immunofluorescence image displaying arrested Yki-driven intestinal stem cell tumors [right; tumors marked in GFP (green)] from adult fruit fly fed TONDU peptide. Untreated tumors populate the entire gut (left).

**What changes do you think could improve the professional lives of early-career scientists?**

Many early-career scientists (ECSs) are either in the last lap of their postdoctoral tenure or probably in a transition phase of their professional life; gearing to embark on an independent research career, build a new interdisciplinary research team, define a new research area, etc. It is essential that ECSs get the right mentorship, good visibility, networking and the right scientific environment that sustains their enthusiasm and nurtures their scientific interests. It would be hugely helpful if they are encouraged to explore unchallenged research areas, and be given support and incentives to pursue challenging and out-of-the-box thinking. This would help ECSs break new grounds and initiate new research areas early in their career and build on them as an independent investigator.

**What's next for you?**

I am now looking forward to setting up my own independent research group and pursuing my interest in developing *Drosophila* disease models, particularly for cancer. Broadly, I am interested in understanding the deterministic role of developmental programs in cancer progression. For example, how many embryonic genes are re-expressed in several human malignancies. It is in this context that I would like to undertake a comprehensive unbiased genetic screen complemented with -omics studies to unravel key host genetic factors that influence the course of epithelial tumor progression. Ultimately, I would like to translate the key insights obtained to design targeted therapeutic strategies, to be later validated in pre-clinical mouse models.
